# Roles of StearoylCoA Desaturase-1 in the Regulation of Cancer Cell Growth, Survival and Tumorigenesis

**DOI:** 10.3390/cancers3022462

**Published:** 2011-05-20

**Authors:** R. Ariel Igal

**Affiliations:** Department of Nutritional Sciences and Rutgers Center for Lipid Research, Rutgers, the State University of New Jersey, 96 Lipman Drive, New Brunswick, NJ 08901, USA; E-Mail: igal@aesop.rutgers.edu; Tel.: +1-(732) 932 9717; Fax: +1-(732) 932 6837

**Keywords:** fatty acid synthesis and desaturation, cancer, cell cycle, apoptosis, lipogenesis, Akt signaling, AMPK, cancer therapeutics

## Abstract

The development and maintenance of defining features of cancer, such as unremitting cell proliferation, evasion of programmed cell death, and the capacity for colonizing local tissues and distant organs, demand a massive production of structural, signaling and energy-storing lipid biomolecules of appropriate fatty acid composition. Due to constitutive activation of fatty acid biosynthesis, cancer cell lipids are enriched with saturated (SFA) and, in particular, monounsaturated fatty acids (MUFA), which are generated by StearoylCoA desaturase-1, the main enzyme that transforms SFA into MUFA. An increasing number of experimental and epidemiological studies suggest that high levels of SCD1 activity is a major factor in establishing the biochemical and metabolic perturbations that favors the oncogenic process. This review examines evidence that suggests the critical implication of SCD1 in the modulation of multiple biological mechanisms, specifically lipid biosynthesis and proliferation and survival signaling pathways that contribute to the development and progression of cancer.

## 1. Introduction

One of the most common traits of cancer is the radical modification of cellular metabolism that promotes and sustains critical features of malignant transformation, *i.e.*, unremitting cell proliferation, increased resistance to programmed cell death and the capacity to invade local tissues and metastasize distant organs. A major aspect of the altered metabolic program of cancer cells is the activation of biosynthetic reactions to supply the rapidly dividing cancer cells with structural, energetic and signaling molecules needed for the generation of daughter cells. Among these routes of macromolecule production, the activation of lipid biosynthesis, particularly *de novo* synthesis of fatty acids, is a major event in the metabolic transformation that leads to cancer [1]. Cancer cells achieve a high rate of fatty acid synthesis by the synchronized activation of the tandem of fatty acid biosynthetic enzymes ATC-citrate lyase (ACL), acetyl-CoA carboxylase (ACC) and fatty acid synthase (FAS), which yield abundant palmitic acid and, by subsequent elongation, stearic acid, the two main saturated fatty acids (SFA) found in these cells. However, a distinctive aspect of this lipogenic transformation is that cancer cells also contain a large pool of monounsaturated fatty acids (MUFA), which are largely generated from SFA by the action of stearoyl-CoA desaturases (SCD). The participation of ACL, ACC and, in particular, FAS in the mechanisms of oncogenic transformation has been extensively explored, as described in excellent reviews [1-4]. The implication of SCD1 in cancer has remained much less investigated. Studies showing that abnormally high levels of SCD1, the best characterized SCD isoform, are commonly found in oncogene-transformed cells and several types of cancer cells provided initial evidence that this enzyme may be functionally associated with the onset and progression of cancer. The crucial involvement of SCD1 in cancer was brought to focus by recent studies that revealed the key roles of this enzyme in the coordinated regulation of metabolic activity and survival signaling in cancer cells. This review describes several lines of evidence, both experimental and epidemiological, that outlines the participation of SCD1 in the oncogenic mechanisms that lead to cancer development.

## 2. Characterization of SCD1 Activity and Distribution

SCD activity, most especially in proliferating cells, is a key factor in promoting a balance of SFA and MUFA in cell lipids that is appropriate to meet the functional demands of the cell. SCDs are Δ9-fatty acylCoA desaturases that catalyze the introduction of a double bond in the cis-delta-9 position of several saturated fatty acylCoAs, mainly palmitoylCoA and stearoylCoA, to produce palmitoleoyl- and oleoylCoA, respectively [5,6]. Mammalian organisms express up to four SCD isoforms, all of which reside exclusively in the endoplasmic reticulum compartment of the cell. SCD1, a main SCD variant found in all mammalians including humans, is present in most tissues and cells, with the highest expression displayed in brain, liver, heart and lung [7]. Other SCDs, like murine SCD2, SCD3 and SCD4, which exhibit high homology with SCD1, are expressed in a predominantly organ-specific manner [8]. Human tissues contain two SCD variants, SCD1 and SCD5. SCD1 expression is remarkably sensitive to a myriad of nutrients, including carbohydrates, fatty acids, and cholesterol, and is regulated by a great number of hormones and growth factors [8]. SCD5, a recently discovered human SCD [6-9] which is also present in chicken, pigs and bovines [10], shows little homology with human SCD1 and other mammalian SCDs [6]. Expression of SCD5 is higher in embryo tissues and in adult human brain and pancreas [8,9]; its biological function, however, remains largely uncharacterized.

## 3. SCD1 is the Main Regulator of Fatty Acid Composition in Cancer Cells

The control of the diversity of lipid species bears fundamental biological relevance in proliferating cells. The wide array of biological functions of phospholipids and their derivatives, such as diacyglycerols, lysophosphatidic acid and phosphatidic acid, displayed during mitogenesis depends on the content of their acyl species, particularly SFA and MUFA [11-13]. A common finding in several cancer cells and tissues is the presence of abnormally high levels of MUFA in all major glycerolipids [14]. Early studies using liver tumors showed evidence of an enrichment of tumor cell lipids with MUFA at the expense of SFA and polyunsaturated fatty acids (PUFA) [15]. More recent work done in cellular models of oncogenic transformation confirmed that abnormally high levels of MUFA is a biochemical hallmark of the carcinogenic process and that this greater MUFA abundance is predominantly caused by elevated SCD1 activity [16-18]. Interestingly, although SFA substrates originating from either exogenous and endogenously sources can be used by SCD1 for the production of MUFA [16], the principal source of substrates for desaturation originates in the massive *de novo* formation of SFA in cancer cells driven by constitutively overexpressed FAS [14]. In these cells, a significant percentage of SFA substrate for SCD1 appears to originate in the highly active glucose metabolism [17], a metabolic hallmark of rapidly proliferating cells [19]. In some cancer cells, glutamine may also contribute with post-mitochodrial metabolites to supply the biosynthesis of fatty acids [20], whose resulting SFA could potentially be used as substrates for the Δ9-desaturating reaction.

The impact of high SCD1 levels in the quality control of the acyl composition of cancer cell lipids is radical and global. Oncogene-transformed and cancer cells display a remarkable enrichment of all major acyl-containing lipids with MUFA [16-18,21,22]. The relevance of SCD activity in defining the fatty acid composition in cancer cells was further exposed by the finding that the inhibition of SCD1 expression/activity leads to a marked decrease in MUFA in cancer cell lipids [17,18,21,22]. The observation that a progressive reduction in SCD1 expression induced in neoplastic cells mirrored a proportionate decay in the content of cellular MUFA established that SCD1 is, indeed, the main modulator of MUFA homeostasis [21]. Importantly, cancer cells appear to have a critical dependence on endogenously synthesized MUFA. This requirement was revealed by data showing that cancer cells with deficient SCD1 attempt to compensate for the reduced supply of endogenous MUFA with an increase in the uptake of exogenous oleic acid [21]. The absence of a sensitive compensatory mechanism for overcoming the harmful effects of deficient MUFA synthesis in these cells is highlighted by the fact that only supraphysiological concentrations of MUFA can restore the biological phenotype of cancer cells undergoing a total suppression of SCD activity [23]. Furthermore, the fact that changing levels of MUFA in cancer cells is followed by an opposing modification in the relative content of PUFA [16,21-23], suggests that an alteration in the levels of this diverse group of acyl molecules with powerful biological functions may also have implications in the phenotype of cancer cells.

## 4. SCD1 Plays a Key Role in the Regulation of Lipid Biosynthesis in Cancer Cells

The functional advantage of the accumulation of MUFA in cancer cells is still not well understood, but evidence points out to the participation of SCD1-derived MUFA in a variety of regulatory mechanisms that are essential for cell proliferation and survival. A major role of SCD1 in mammalian cells is the regulation of lipid synthesis. Hulver *et al.* [24] demonstrated that an increase in SCD1 in human myoblasts was sufficient to enhance the formation of phospholipids and triacylglycerols. Conversely, in oncogene-transformed cells and cancer cells, it was observed that abrogation of SCD1 expression/activity promoted a marked decrease in the *de novo* synthesis of phospholipids, including the main membrane polar lipids phosphatidylcholine and phosphatidylethanolamine [17,18,21,22], confirming that SCD1 plays a critical role in the overall production of acyl-containing lipids. Interestingly, suppression of SCD activity did not noticeable modify the content of cell phospholipids [23], suggesting that, as a balancing mechanism to maintain steady levels of membrane lipids, degradation of phospholipids may be downregulated in a state of SCD deficiency. Data showing that SCD1 activity also governs the rate of biosynthesis of triacylglycerol and cholesterolesters [17,18,21,23], which are usually found in low concentrations in cancer cells, gives support to the notion that SCD1 is a central point of regulation in the lipogenic program in cancer.

Research work over the past few years indicates that SCD1 may control the overall rate of cancer cell lipogenesis by at least three major mechanisms involving substrate availability for lipid biosynthesis, metabolic control of fatty acid biosynthesis, and control of growth and survival signaling. As described above, SCD1 determines the balance of MUFA and SFA, which are the most abundant substrate pools for acylation reactions in the cancer cell. It has been established that acyltransferases preferentially incorporate MUFA into newly synthesized lipids than SFA [25,26]; hence, a chronically up-regulated SCD1 may be able supply the overactive lipid biosynthetic machinery of fast-replicating cancer cells with a great provision of ideal fatty acid substrates. Moreover, SCD1 may contribute to propel lipogenesis by enhancing fatty acid synthesis through mechanisms involving the preservation of catalytically active ACC, a key enzyme that catalyzes the formation of malonylCoA in the fatty acid biosynthetic pathway. It was shown that high SCD1 contributes to suppress the activity of AMPK [17], which mainly targets ACC for inactivation through hyperphosphorylation. By steadily converting SFA into MUFA, SCD1 may also maintain ACC catalysis in a state of persistent activation through the continuous removal of saturated acylCoAs, the most powerful allosteric inhibitors of ACC [27-29]. Finally, recent findings revealed that SCD1 activity is required for the full activation of the Akt pathway [18,22]. As part of their pro-growth, pro-survival activity, Akt-derived signals control the transcriptional activation of a number of lipogenic enzymes [1,20]; therefore, a constitutively active SCD1 may also favor a lipogenic phenotype in cancer cells by maintaining a high activation rate of Akt. In summary, the excess MUFA biosynthesis produced by high SCD1 is not just a byproduct of metabolic transformation but an influential factor in the dual control of global lipid biosynthesis and survival signaling cascades which, as it is described below, will ultimately modulate central biological mechanisms in cancer cells.

## 5. Control of Cell Cycle and Proliferation Rate by SCD1

In order to multiply, rapidly dividing cells, particularly cancer cells, must activate the production and remodeling of lipid signals and structural lipid macromolecules [30]. Previous studies demonstrated that the synthesis of major lipid bricks, like SFA, and membrane-forming macromolecules, such as phospholipids and cholesterol, are coordinately regulated with cell cycle [30-32]. However, cells must expand the amount of lipids with a fatty acid composition that is appropriate for maintaining the functions of dividing cells which, in case of fast-duplicating cells, must contain substantial amounts of MUFA. The implication of SCD1 in the mechanisms of cell mitogenesis was first suggested by the finding that potent cytokines that activate cell cycle, such as platelet-derived growth factor (PDGF), epidermal growth factor (EGF) and insulin, among several others, induce SCD1 expression in a number of human cell types [33,34]. Elegant studies conducted by Jean-Baptiste Demoulin's group demonstrated that the mitogen-induced up-regulation of SCD1 expression is parallel to the activation of the synthesis of phospholipids and cholesterol [33], underscoring the critical involvement of MUFA synthesis in the coordinated activation of lipogenesis during cell cycle.

Recently released information offered additional proof to the notion that SCD1 is not only a target for cell cycle-regulating mechanisms but a relevant player in the molecular events involved in cell division. Studies in cancer cells showed that SCD activity controls the passage of cycling cells through the G1 phase into S phase [18,23]. The observation that FAS and ACC activities are also fundamental factors in similar stages of cell cycle progression [23,35,36] implies that a coordinated activation of SFA synthesis and its conversion to MUFA must take place in order to enter the synthetic phase cell cycle. This argument is further supported by the demonstration that cell cycle blockade produced by both ACC and SCD1 inhibitors is overcome by exogenously added MUFA [23], again indicating that the fatty acid biosynthetic pathway of cancer cells produces key molecules, likely MUFA, that facilitates the progression of cell cycle. Moreover, the fact that phospholipid biosynthesis and remodeling occur during G1 and early S-phase [30,31] further denotes the great deal of coordination between MUFA production and the phospholipid biosynthetic machinery during specific phases of the cell cycle. Finally, although the molecular mechanisms by which SCD1 influences cell cycle are largely unknown, recent data indicate that SCD1 may control cell cycle by altering the levels of cyclin D1 and CDK6 [23], two proteins involved in the progression of cycling cells through G1 phase [37].

## 6. SCD1 is an Essential Anti-Apoptotic Factor

Early studies from Roger Unger's lab revealed the negative impact of excess free fatty acids on the functionality of pancreatic β-cells, coining the term “lipotoxicity” to describe this lipid-mediated cytotoxic phenomenon [38]. Few years later, DeVries *et al.* [39] observed that excess SFA induce a similar cytotoxic effect in cardiomyocytes, whereas MUFA exhibited a neutral biological effect. This ubiquitous deleterious effect of SFA, predominantly driven by accumulation of palmitic and stearic acids, was reported in various cells and tissues, including CHO cells [40], β-pancreatic cells [41-43], breast cancer cells [44], as well as in whole liver and pancreas [45,46]. Since the source of excess fatty acids in these studies was external, the authors postulate the idea that an abnormal increase in circulating fatty acids, particularly SFA, will trigger an initial stress response in targeted cells, followed by entry in the apoptotic program if these fatty acids cannot be rapidly segregated into metabolic pathways that will render less harmful lipid molecules.

As mentioned earlier, a biochemical hallmark of cancer cells is the constitutive activation of SFA production; hence, in order to avoid its detrimental accumulation, this surplus of endogenous SFA needs to be efficiently managed by the cancer cell. In these cells, the presence of a highly active SCD1 may be a crucial metabolic requirement to avert the stressful impact of SFA by processing these fatty acids into more biologically neutral MUFA. In support of this postulate, it was reported that ablation of SCD1 expression and activity in cancer cells activates the program for cell suicide, likely as the result of intracellular SFA buildup [17,21,23]. Importantly, this SCD1-mediated safeguard mechanism for preventing lipotoxic damage is also present in non-transformed cells, likely to defuse the potential damage of an eventual overload of palmitic acid in plasma [47].

Although the molecular mechanisms by which excess SFA trigger an immediate stress response that eventually evolves into cell apoptosis are still unknown, evidence points out to perturbations in the processes of phospholipid synthesis and remodeling, as well as in triacylglycerol homeostasis. It has been reported that, by decreasing the ratio MUFA to SFA in cell phospholipids, the inhibition of SCD1 expression induces the activation of unfolded protein response [48,49], a preliminary step in the cell suicidal program. In an attempt to compensate for the deficient acylation of MUFA in phospholipids, SCD1-depleted cells up-regulate the expression of lysophosphatidylcholine acyltransferase-1 and, especially, lysophosphatidylcholine acyltransferase-3, which preferentially incorporates polyunsaturated fatty acids into phosphatidylcholine [48], suggesting that the enrichment of this phospholipid with saturated species is responsible, at least partly, for initiating the program of apoptosis in SCD1-deficient cells.

The alterations in molecular species of membrane phospholipids arising from SCD1 deficiency may also impair the function of organelles where signals for programmed cell death are initiated. Accumulation of SFA in phospholipids caused by suppression of SCD1 or by excess exogenous palmitate promotes the disruption of endoplasmic reticulum morphology and the activation of stress response in this subcellular compartment [50,51]. Similar metabolic conditions lead to alteration in the synthesis of cardiolipin, a lipid that plays essential roles in mitochondria [52]. The mechanisms of cytotoxicity triggered by SCD1 deficiency also appear to involve a dysfunctional metabolism of diacylglycerol and ceramides, two critical phospholipid-derived signaling lipids that take part in cell stress response and programmed cell death [53].

The participation of triacylglycerol metabolism in the mechanisms of cell stress and apoptosis in mammalian cells and tissues has been well documented [47]. The presence of an expandable lipid pool, such as triacylglycerols, in mammalian cells is a vital metabolic barrier against fatty acid-mediated apoptosis [40,54]. In cancer cells, although triacylglycerols represent a quantitatively minor lipid, the regulation of content and fatty acid composition of this lipid fraction may be critical for maintaining cell viability. In conditions of SCD1 depletion, neoplastic cells force the segregation of SFA away from phospholipids and into triacylglycerols [21], in a possible attempt to preserve, albeit temporarily, a membrane acyl composition that is compatible with cell proliferation. However, this adaptation response cannot be sustained over time and triacylglycerol homeostasis is disrupted by the excess accumulation of SFA-enriched species, initiating the lipotoxic response that culminates in apoptosis [40,54].

## 7. Modulation of Survival Signaling by SCD1

As aforementioned, the changes in fatty acid distribution and in overall lipid biosynthesis promoted by SCD1 appear to be crucial to promote and sustain typical traits of malignant behavior, such as high rate of cell proliferation and cell survival. Recent studies indicate that changing levels of SCD activity may have significant implications in the response of cancer cells to stimuli that activate mitogenic and survival signaling pathways. Studies done in lung cancer cells and, more recently, in prostate cancer cells revealed that SCD1 is a key modulator of the phosphatidylinositol-3 kinase/Akt pathway, a central signaling cascade involved in the regulation of lipid biosynthesis, growth and replication of mammalian cells [18-22]. The induction of lipid biosynthesis triggered by Akt activation appears to be mediated through a mechanism involving the activation of the sterol response element binding protein-1 (SREBP-1) [55], a transcriptional factor that is a main controller of lipogenesis [56]. SCD1 is known to influence SREBP-1 functional activation; hence, it is possible that the regulatory mechanism by which SCD1 affects lipid production through modulation of Akt requires the participation of SREBP-1.

Besides its impact on the rate of lipid biosynthesis, the influence of SCD1 activity on Akt signaling cascade could also be an important determinant in other mitogenic mechanisms, such as the regulation of cell cycle. It has been observed that suppression of SCD1 leads to dephosphorylation and activation of glycogen synthase-kinase β (GSK3β), a downstream component of Akt pathway that inhibits cell proliferation by increasing the degradation of cyclin D1 [57]. The finding of low levels of cyclin D1 detected in cancer cells undergoing a blockade of SCD1 [23] suggests a direct link between reduced Akt activity with a subsequent activation of GSK3-β and the deregulation of cell cycle progression observed in these cells.

The mechanistic components that integrate SCD1 activity, growth and survival signaling pathways, and their downstream effectors remain unidentified. However, it is conceivable that SCD1 activity, by controlling the acyl composition of membrane phospholipids, modulates, either sequentially or concurrently, the activation rate of plasma membrane-resident signaling platforms linked to the Akt cascade, such as growth factor-activated tyrosine kinase receptors. In this hypothetical scheme, an overly active SCD1 could contribute to the overactivation of mitogenic signaling cascades associated to epidermal growth factor receptor (EGFR), insulin and insulin-like growth factor receptors, typically observed in a number of cancers [58]. Although direct evidence supporting this hypothetical mechanism is lacking, *in vitro* studies indicate that phospholipids enriched with MUFA induce activation of EGFR by triggering tyrosine autophosphorylation [59], whereas saturated phosphatidylcholine produces the opposite effect [60]. Changing levels of SCD1 are known to affect the activity of ABC-A1 transporters in plasma membrane [61], but a similar regulation of cytokine-activated receptors by the desaturase has yet to be demonstrated. Future cell-based studies will provide more definitive answers to the question of whether the greater MUFA-to-SFA ratio in membrane phospholipid species directly affects the induction of signaling cascades by growth factors in cancer cells.

Finally, defined plasma membrane microdomains, such as raft-like and non-raft structures, are fundamental for proper mobility and activation of tyrosine-kinase receptors and their immediate downstream effectors [62,63]. The lipid composition of these domains is known to influence the response of these signaling platforms to cytokine stimulus [63,64]. Given the predominance of SFA and MUFA in cancer cell lipids, SCD1 activity may be a crucial factor in controlling the dynamics of plasma membrane microdomains and, consequently, the function and compartmentalization of protein complexes that reside and interact in these domains. This hypothetical mechanism of signaling regulation, based on microenvironmental control by SCD1, awaits empirical confirmation.

## 8. Implication of SCD1 in the Onset and Progression of Cancer: Experimental and Epidemiological Evidence

A growing amount of data gathered from experimental studies and epidemiological reports suggests that abnormal SCD1 activity may be positively associated with several types of cancers. Overexpression of SCD1 has been observed in colonic and esophageal carcinoma and in hepatocellular adenoma [65], in hepatocarcinoma [66,67], as well as in carcinogen-induced tumors in rodents [68,69]. Importantly, a recent comprehensive investigation using mouse prostate cancer models and human prostate cancer tissue demonstrated that both MUFA content and SCD1 expression are elevated during the progression of cancer from benign hyperplasia to cancerous epithelium [18]. Another recently published study identified SCD1 gene as a high BMI prostate cancer signature [70]. However, despite these findings, there is still some discrepancy regarding the potential association of high SCD1 expression and prostate cancer [71]. This disagreement could be attributed to different experimental and technical approaches employed to assess the presence of SCD1 in prostate cancer samples.

Epidemiological studies also suggest a positive correlation between high levels of SCD1 expression and activity and the presence of cancer. It was found that low stearic acid content with high oleic acid levels in sera from breast cancer patients were positively linked to cancer appearance [72,73]. Moreover, a relationship between abnormal distribution of SFA and MUFA in erythrocyte membranes and breast cancer risk was reported [74]. Other studies show that high MUFA levels were correlated with poor prognosis and a greater cancer death rate in patients [75-79]. These epidemiological observations draw a connection with findings in *in vitro* studies described above in which expression/activity of SCD1 was reportedly elevated in cancer cells, suggesting that overexpressed SCD1 could likely be the cause of the aberrant fatty acid composition in cancer patients. However, in an excellent review of epidemiological studies, Veronique Chajes *et al.* [79] postulate the intriguing notion that, besides the role of SCD1 up-regulation in cancer cells, changing levels of SCD1 expression/activity in liver may be related to breast cancer onset, suggesting that SCD1 may play a broader role in the tumorigenic mechanisms, likely participating in setting up the appropriate environmental conditions for the onset of cancer.

Supporting the aforementioned descriptive studies that propose a link between SCD1 deregulation and development of cancer, recent loss-of-function studies in cells demonstrated the active role of SCD1 in the regulation of carcinogenesis. In both oncogene-transformed cells and tumor-forming lung cancer cells, it was shown that the stable silencing of SCD1 expression was able to reduce overall lipogenesis, impair cell proliferation and trigger cell death mechanisms [17,18,21-23]. Active SCD1 appears to be a rather universal factor in survival mechanisms since ablation of SCD1 promotes an anticancer effect in a wide variety of neoplastic cells [14,18,48]. The essentiality of constitutive MUFA synthesis for the growth and survival of different cancer cell types is also supported by the fact that these cells express an ample range of SCD1 expression and show a comparable reduction in cell proliferation in conditions of SCD1 depletion. Furthermore, the observation that, unlike cancer cells, normal human cells subjected to a prolonged inhibition of SCD1 activity do not significantly affect their rate of proliferation [17,23,49], indicates that SCD1 plays an indispensable mitogenic role in rapidly replicating cells.

Besides its participation in the mitogenic mechanisms of cancer cells, SCD1 participate in the modulation of the tumor formation process. Falvella *et al.* [68] reported that the background level of SCD1 expression is associated with genetic predisposition to hepatocarcinogenesis in mice and rats. These authors found that the rate of cancer appearance was greater in rodents with higher levels of SCD1 than in strains with low SCD1 expression. More definitive evidence on the participation of SCD1 in tumorigenesis was provided by experiments using xenograft tumor models originated from lung cancer cells, in which the rate of tumor appearance and growth was severely affected when SCD1 expression was blocked [22]. A more recent study in a murine prostate cancer model also showing a tumor-suppressing effect of SCD1 blockade adds further support to the argument that highly functional SCD1 is a critical factor in the mechanisms of tumor growth [18]. Although experimental and epidemiological research implies a function for SCD1 in cancer development, whether SCD1, as it occurs with FAS [1,3], is a common feature of most cancer types, as well as a biomarker for disease prognosis, cannot be inferred from past studies; thereby, more basic research work, specifically large-scale histopathological studies, and more decisive epidemiological studies will provide a more definitive answer.

## 9. Conclusions

**Figure 1.**
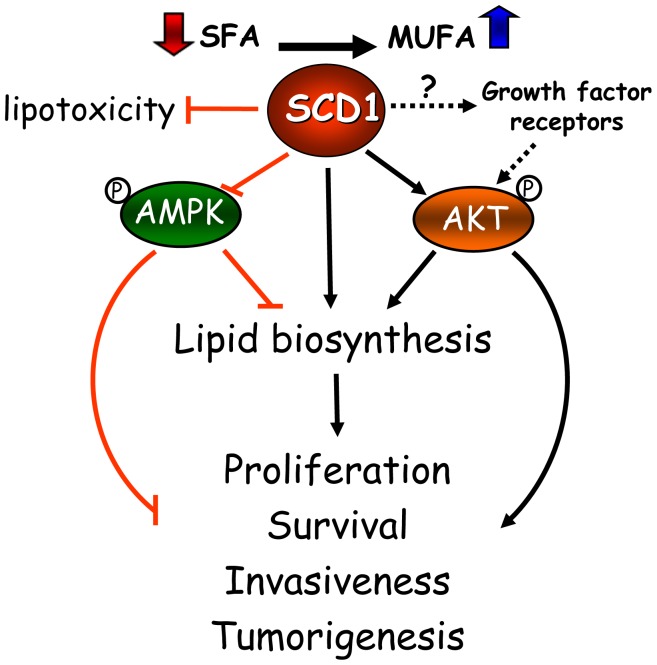
A hypothetical model for metabolic and signaling control by SCD1 in cancer. AMPK, AMP-activated kinase; MUFA, monounsaturated fatty acids; SFA, saturated fatty acids; SCD1, Stearoyl-CoA Desaturase 1.

Based on this increasing collection of cell-based and animal tumor studies, as well as in epidemiological observations in cancer patients, SCD1 has emerged as a critical factor in the mechanisms of cancer onset and progression. These studies have revealed the central role of the desaturase in a series of interconnected metabolic and signaling pathways that support the biochemical and biological phenotype of cancer cells (Figure 1). SCD1 appears to modulate multiple pathways and cascades in cancer cells, including fatty acid biosynthesis through the regulation of ACC, either by allosteric and/or AMPK-mediated mechanisms; glycerolipid formation, likely through the enhanced production of MUFA substrates to the acyltransferases; and the Akt signaling pathway, potentially by affecting the activation status of signaling proteins belonging to this pathway. Whether the regulation of signaling events precedes the changes in global lipogenesis induced by the desaturase has yet to be determined. Additionally, evidence obtained from lipotoxicity cell models highlights another not less fundamental role of SCD1 activity in cancer cells: its ability to suppress a potentially toxic accumulation of SFA by its simple conversion into MUFA, averting cell stress response and subsequent apoptosis.

Finally, the findings described here support the concept that SCD1 may be a potentially useful druggable target for new cancer treatments. An increasing number of specific small molecule inhibitors of SCD1 activity have been recently synthesized [80-85], holding a great potential for exploitation as therapeutic agents. It is also tempting to speculate that the association of SCD1 inhibitors with nutritional interventions (*i.e.*, high SFA diets) and/or therapeutic agents that target signaling pathways and their receptors (*i.e.*, tyrosine-kinase-mediated cascades, such as EGFR, Her2, IGF-1, among others) may originate more effective anti-cancer treatments. Despite these hypothetical considerations, establishing the value of SCD1 inhibitors as preventive or treating agents in different forms cancers will require more extensive experimental testing and careful pre-clinical validation.

## Abbreviations

ACC: acetylCoA carboxylase
AMPK: AMP-activated kinase
FAS: fatty acid synthase
MUFA: monounsaturated fatty acids
PI3K: phosphatidylinositol-3 kinase
SCD: StearoylCoA desaturase
SFA: saturated fatty acid
SREBP: sterol response element binding protein
